# Parametric Investigation and Optimization to Study the Effect of Process Parameters on the Dimensional Deviation of Fused Deposition Modeling of 3D Printed Parts

**DOI:** 10.3390/polym14173667

**Published:** 2022-09-03

**Authors:** Muhammad Abas, Tufail Habib, Sahar Noor, Bashir Salah, Dominik Zimon

**Affiliations:** 1 Department of Industrial Engineering, University of Engineering & Technology, Peshawar 25100, Pakistan; 2 Industrial Engineering Department, College of Engineering, King Saud University, P.O. Box 800, Riyadh 11421, Saudi Arabia; 3 Department of Management Systems and Logistics, Rzeszow University of Technology, 35-959 Rzeszow, Poland

**Keywords:** fused deposition modeling, polylactic acid (PLA), dimensional deviation, definitive screening design, desirability function

## Abstract

Fused deposition modeling (FDM) is the most economical additive manufacturing (AM) technology available for fabricating complex part geometries. However, the involvement of numerous control process parameters and dimensional instabilities are challenges of FDM. Therefore, this study investigated the effect of 3D printing parameters on dimensional deviations, including the length, width, height, and angle of polylactic acid (PLA) printed parts. The selected printing parameters include layer height, number of perimeters, infill density, infill angle, print speed, nozzle temperature, bed temperature, and print orientation. Three-level definitive screening design (DSD) was used to plan experimental runs. The results revealed that infill density is the most consequential parameter for length and width deviation, while layer height is significant for angle and height deviation. The regression models developed for the four responses are non-linear quadratic. The optimal results are obtained considering the integrated approach of desirability and weighted aggregated sum product assessment (WASPAS). The optimal results include a layer height of 0.1 mm, a total of six perimeters, an infill density of 20%, a fill angle of 90°, a print speed of 70 mm/s, a nozzle temperature of 220 °C, a bed temperature of 70 °C, and a print orientation of 90°. The current study provides a guideline to fabricate assistive devices, such as hand and foot orthoses, that require high dimensional accuracies.

## 1. Introduction

Among the AM technologies, fused deposition modeling (FDM) is one of the most widely used additive manufacturing technologies because of its economy and ability to process a diverse range of materials, including polymers and metals [1]. However, the use of FDM printing for part fabrication is still a challenge because of the involvement of numerous process parameters and because the choice of materials affects the part quality, mechanical strength, and development time [2,3]. Depending on the application, careful consideration of process variables and material selection is necessary. According to the published reports, the process parameters can be divided into three major sets [4]. The first set of parameters includes the process-related parameters, such as infill speed, number of shells, thickness of shells, bed temperature, fill density, layer height, nozzle temperature, print speed, air gap, and raster angle. The second set of parameters includes the machine-specific parameters, such as nozzle diameter, filament width, bed adhesion type, and filament diameter. The third set of parameters is related to part geometry, such as the part’s orientation and special features.

To achieve good dimensional accuracy in FDM-printed parts, the optimal process parameter settings are crucial, as they vary according to material, complexity of part geometry, material type, and chemical composition [5,6]. Therefore, finding the optimal settings and combination of parameters can be challenging and laborious. Additionally, most of the polymers used in FDM are semi-crystalline and prone to part distortion due to crystallization [7]. Therefore, the process requires trial and error experimental procedures, or application of the design of experiments (DoE), to achieve excellent quality prints with desirable mechanical properties. The most common semi-crystalline polymers are polylactic acid (PLA), polypropylene (PP), polycaprolactone (PCL), polyethylene (PE), and polybutylene terephthalate (PBL). Moreover, the dimensional specifications may vary for the same material as well as for varied materials. For instance, in PLA, positive deviation (expansion) is observed in the width and thickness direction, while negative deviation (shrinkage) is observed in the length direction [8].

PLA is considered a green material because it is made through the polymerization of lactic acid by the fermentation of renewable resources. There are four different forms of crystals, namely α, β, and γ [9]. The α crystals show two disordered modifications i.e., $\alpha {}^{\prime }$ and $\alpha {}^{\prime \prime }$ [9]. The α crystal is obtained through cold, melt, or solution crystallization at a higher temperature (i.e., above 120 °C) [10], while $\alpha {}^{\prime }$ is produced at a lower temperature (i.e., below 100 °C) by mixing α and $\alpha {}^{\prime }$ between 100 °C and 120 °C [11]. The $\alpha {}^{\prime \prime }$ crystal is obtained through crystallization at a temperature (0 °C to 30 °C) under high-pressurized CO_2_ [12]. The $\alpha {}^{\prime }$ crystal forms the chain conformation of the PLA chain, which is more disordered than in the α form crystal [13]. Therefore, the α form provides lower elongation at break, higher Young’s modulus, and better preservation against water vapor than the $\alpha {}^{\prime }$ form. The $\alpha {}^{\prime \prime }$ crystal produces poor chain packing and the lowest crystal density compared to α and $\alpha {}^{\prime }$ [14]. The published studies have shown that the α form crystal is more stable compared to its other forms [9]. The β form crystal is obtained through α crystal deformation and through annealing or stretching at elevated temperatures [15]. The γ form is obtained by epitaxial growth on a hexamethyl benzene substrate [16].

The physical and mechanical properties of PLA are influenced by the degree of crystallinity. Mechanical properties can be improved by thermal annealing to increase the degree of crystallinity [17]. In FDM printing, the degree of crystallinity in the bottom layers is higher than in the top and side layers because of the bed temperature, which causes the layer to cool down slowly, thus rendering the printed part dimensionally unstable [18].

## 2. Literature Review

Numerous studies have been reported that investigated the effect of FDM process parameters on quality characteristics, mechanical properties, physical properties, energy consumption, and build time for diverse types of materials. For instance, Galetto et al. [4] investigated the effect of process parameters on the process efficiency and quality of PLA printed parts. Quadratic models were developed for surface roughness and dimensional accuracies. For maximizing dimensional accuracy, the design features of parts play a significant role. Kitsakis et al. [19] studied the dimensional accuracy of FDM-printed parts for medical applications. In the study, they considered different parameters, including the material type (PLA and ABS), layer height, infill rate, and the number of shells, as well as studying the dimensional accuracy. The study revealed that the best dimensional accuracy for PLA material was attained at an infill rate of 50%, with one shell, and a layer height of 0.3 mm. The study of Aslani et al. [20] showed that the extrusion temperature significantly affects the dimensional accuracy and surface roughness of PLA printed parts. The study proved that by applying grey relational analysis, high extrusion temperature (230 °C) combined with medium wall thickness values (2 mm) optimized both surface roughness and dimensional accuracy. Nathaphan and Trutassanawin [21] concluded that for good dimensional accuracy and compression strength, the layer height and print speed must be set at a low level, the nozzle temperature at a high level, while the bed temperature must be above the glass transition temperature of ABS material. Further, shrinkage occurs in the diameter of the cylinder because of the cooling and solidification of molten polymer. However, expansion was noticed in height of the cylinder due to the rounding of the number of layers to the higher integer number. Basavaraj and Vishwas [22] found that layer thickness affects the tensile strength, manufacturing time, layer thickness, shell thickness, and orientation angle. Further, the study concluded that tensile strength and dimensional accuracy decrease with an increase of the layer thickness and increase with increases of the orientation angle and shell thickness. The study of Lalegani Dezaki et al. [23] revealed that surface quality and mechanical properties are directly affected by the type of patterns. Concentric and grid patterns exhibit good surface quality and tensile strength while the zigzag pattern produces the worst surface roughness and mechanical properties. Padhi et al. [24] noted that shrinkage occurs along the width and length directions, while the thickness increases in parts printed from acrylonitrile-butadiene-styrene (ABSP 400). The shrinkage may develop inner stress upon solidification. Further, the formation of inner layer cracks and weak interlayer adhesion decrease the dimensional accuracy of final parts. Vahabli and Rahmati [25] improved the surface quality of FDM-printed parts for medical devices using artificial neural networks based on the feed-forward back propagation (FFBP) algorithm. Parts were printed from ABSplus material. The successful fabrication of medical devices such as a molar tooth, femur, skull, and stem further confirms the performance of FFBP. Deswal et al. [8] worked on FDA process parameters by applying an approach integrated with a response surface methodology, artificial neural network-genetic algorithm (ANN-GA), genetic algorithm (RSM–GA), and artificial neural network (ANN) for improving the dimensional accuracy of ABS parts. The adaptive neuro-fuzzy inference system (ANFIS) model and whale optimization algorithm (WOA) was applied by Sai et al. [26] to optimize the process parameters for printing PLA implants. Their study concluded that layer thickness followed by raster angle and infill density significantly affects the surface roughness, while layer thickness and raster angle at low level and infill density at medium level provides good surface quality. The findings of Vyavahare et al. [27] revealed that layer thickness and build orientation have a significant effect on fabrication time and surface roughness, while for dimensional accuracy, in addition to these two parameters, Camposeco-Negrete [28] optimized the process parameters to improve the dimensional accuracy, energy consumption, and the production time of FDM 3D printed acrylonitrile styrene acrylate (ASA) parts. The study showed that printing plane is the most significant parameter that helps in reducing production time and energy consumption. For dimensional accuracy, the infill pattern influences the width of the part, and layer thickness affects the length of the part significantly. Mohamed et al. [29] applied a deep neural network to analyze and optimize the dimensional accuracy of FDM PC-ABS printed parts. In the study, a total of 16 experiments were planned based on a definitive screening design (DSD). The part profile for dimensional accuracy was considered as the percentage variation in diameter and length. The quadratic model was found to be significant for both length and diameter variation. Slice thickness, print direction, interaction of print direction, and deposition angle were found to be significant for length variation. Mohanty et al. [30] applied the hybrid approach of a Taguchi- MACROS- nature-inspired heuristic optimization technique to optimize parameters affecting the dimensional precision of ABS M30 FDM-printed parts. Their results showed that part orientation significantly affected dimensional precision. All of the nature-inspired algorithms considered in the study provide comparable results for minimizing dimensional error. Garg et al. [31] studied the dimensional accuracy and surface roughness of ABS P430 FDM-printed parts under the cold vapor technique using acetone. The results revealed that chemical treatment reduces surface roughness and improves the dimensional accuracy of the final part. This may be attributed to softening of the external layer, because acetone causes rupturing of a secondary bond between the chains of ABD polymers and reaches a more stable position.

The literature review presented above shows that limited studies are available in the literature that focus on the investigation of the effect of different process parameters on dimensional accuracy or dimensional deviation (along the length, width, and height) of FDM-printed parts. According to the best knowledge of the authors, no similar study has been published before concerning angular deviation. Parameters such as the number of shells, bed temperature, infill density, build orientation, and printing speed are studied far less compared to other parameters such as the layer height, infill angle, and extrusion temperature. Therefore, further research is needed to determine the impact of various process parameter combinations on dimensional deviation. Thus, the present study aims to cover the research gaps and offers an inclusive guide for additive manufacturing users to decide on optimal FDM process parameter settings that affect dimensional deviations. Furthermore, an integrated approach of desirability function and weighted aggregated sum product assessment (WASPAS) is proposed for simultaneous optimization of responses.

## 3. Materials and Methods

**Figure 1:**
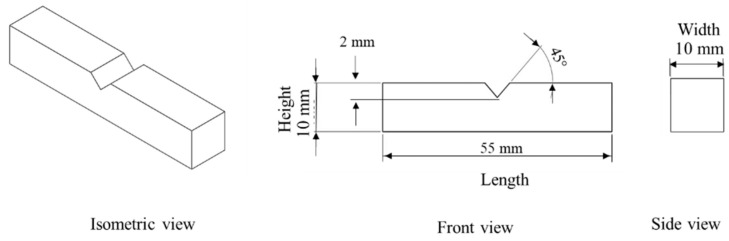
The geometry of the test specimen.

**Figure 2:**
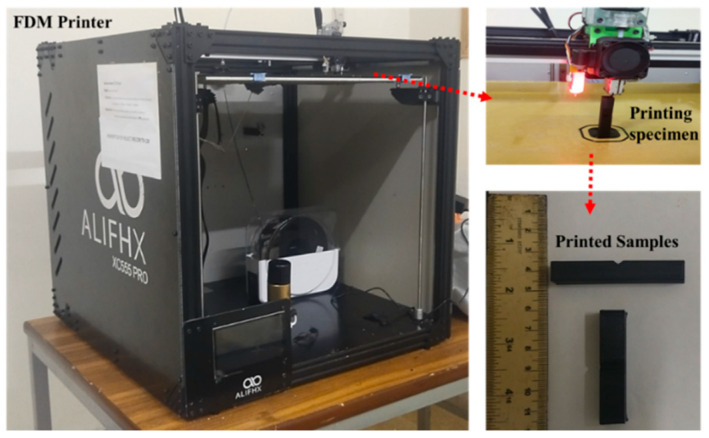
FDM 3D printer, specimen printing, and test samples.

Test specimens were printed from commercial-grade poly lactic acid (PLA) supplied by a local manufacturer (3Dworld, Rawalpindi, Punjab, Pakistan) using an ALIFHX XC555 PRO3D printer. The diameter of the filament is 1.75 mm, having a density and a molecular weight of 1.3 g/cm^3^ and 4.7–16.8 × 10^3^ g/mol. The printed test specimens were prepared according to ASTM E23-12c, which is used for impact tests, as shown in Figure 1. Figure 2 shows the printing system used for the specimens.

**Figure 3:**
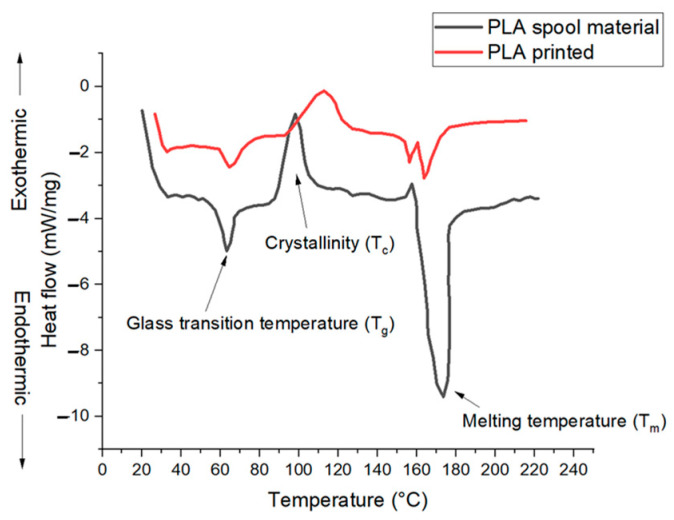
Comparison of differential scanning calorimetry (DSC) of PLA spool material and printed PLA.

Differential scanning calorimetry (DSC) was performed for both PLA spool material and printed PLA, as shown in Figure 3. The three key features of semi-crystalline thermoplastic PLA material represented are the heat flow at a glass transition temperature (T_g_), the cold crystallization exothermic (T_c_), and the melting temperature endothermic (T_m_). The T_g_, T_c_, and T_m_ for spool material are 63 °C, 98 °C, and 170 °C, which agrees with the range of reported values in the literature [32]. For PLA printed material, the T_g_ increases slightly from 63 °C to 65 °C, and melting temperature decreases with the formation of two added peaks i.e., at 164 °C and 157 °C. This may be attributed to the formation of multiple crystalline forms, namely the α and $\alpha {}^{\prime }$ during the thermal cycling [33].

### 3.1. Printing Process Parameters

**Figure 4:**
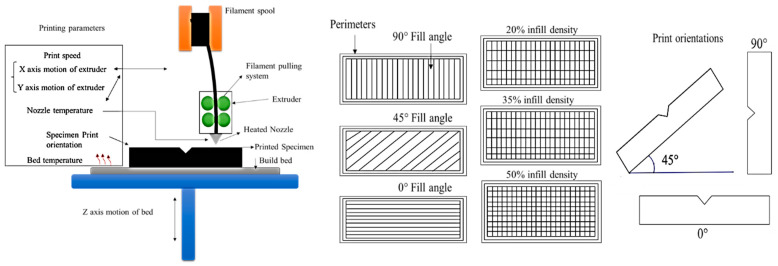
Schematic of FDM 3D printer with associated printing parameters and print orientations.

The printing process parameters investigated in this study include the layer height, number of perimeters, infill density, infill angle, printing speed, nozzle temperature, bed temperature, and print orientation. Figure 4 shows the schematics of FDM printing and selected control printing parameters. For each process, parameter values at three levels were set based on the literature review and recommendation of the material manufacturer, as tabulated in Table 1. The other parameters were kept constant (given in Table 2).

### 3.2. Experimental Design and Measurement of Responses

Due to a large number of process parameters, a systematic experimental design approach, namely the three-level definitive screening designs (DSD) is used to plan experimental runs. The purpose of using this design is to model and estimate the main effect, interaction effect, and quadratic effect in small experimental runs. A total of 17 experimental runs were designed. However, to consider the repeatability of the printing process, the experimental runs were replicated three times randomly. The final experimental design includes 51 experimental runs, tabulated in Table A1 in the Appendix A. The samples prepared according to the experimental design are shown in Figure 1.

The responses considered for the dimensional deviations include the length, width, height, and angle, as shown in Figure 1. The deviation is calculated based on the percentage variation of CAD geometry and printed geometry by using Equation (1). For this, a profile projectile (Mitutoyo PJ-A3000, Mitutoyo Corporation, Kanagawa, Japan) is used. The resolution of the instrument for linear dimensions and angle is 0.01 mm and 0.01°, respectively.

(1) $$\Delta \;X\;\left(\%\right)=\left(\frac{X_{c}-X_{e}}{X_{c}}\right)\times 100$$

where ΔX is the deviations in dimensions, $X_{c}$ is CAD dimensions, and $X_{e}$ is the dimension of a printed specimen.

### 3.3. Optimization Methodology

Responses were optimized individually as well as simultaneously. Single responses were optimized considering the desirability function. To minimize and maximize the response variable, the desirability function is used, which is expressed in Equations (2) and (3).

(2) $$d_{i}(k)\;=\;[\begin{array}{cc} 0, & y_{i}\left(k\right)\;\leq \;\min\left(y_{i}(k\right))\;,\\ {\left[\frac{y_{i}\left(k\right)-\min\left(y_{i}\left(k\right)\right)}{\max\left(y_{i}\left(k\right)\right)-\;\min\left(y_{i}\left(k\right)\right)}\right]}^{r} & \min\left(y_{i}(k\right))\;\leq y_{i}\;\leq \;\max\left(y_{i}\left(k\right)\right)\\ 1, & y_{i}\left(k\right)\;\geq \;\min\left(y_{i}(k\right)) \end{array}]$$

(3) $$d_{i}(k)\;=\;[\begin{array}{cc} 0, & y_{i}\left(k\right)\;\leq \;\min\left(y_{i}(k\right))\;,\\ {\left[\frac{y_{i}\left(k\right)-\max\left(y_{i}\left(k\right)\right)}{\min\left(y_{i}\left(k\right)\right)-\;\max\left(y_{i}\left(k\right)\right)}\right]}^{r} & \min\left(y_{i}(k\right))\;\leq y_{i}\;\leq \;\max\left(y_{i}\left(k\right)\right)\\ 1, & y_{i}\left(k\right)\;\geq \;\max\left(y_{i}(k\right)) \end{array}]$$

where $d_{i}\left(k\right)$ is the desirability value of each response at the *i*th experiment and *k*th response, $y_{i}(k$) is the individual value of measured response k at experiment number *i*, $max\;y_{i}\;\left(k\right)$ and $min\;y_{i}\left(k\right)$ are the maximum and minimum values of data obtained for the *k*th response, and r is the weight of the desirability function.

Simultaneous optimization of responses was performed based on the proposed integrated approach of desirability function and weighted aggregated sum product assessment (WASPAS) method. It transformed the multi-response optimization problem into a single response called a relative importance score. The following procedure was adopted to optimize the process parameters:

*Step 1:* Compute the desirability function for responses using Equations (2) and (3). For minimization (cost criteria), apply Equation (2), while for maximization (benefit criteria) use Equation (3).

*Step 2:* Calculate the weighted sum of desirability functions (*WSD*) using Equation (4).

(4) $$WSD_{i}=\sum_{j=1}^{r}d_{i}w_{j}$$

where $w_{j}$ stands for the weight of jth response.

*Step 3:* Calculate the weighted product of desirability functions (*WPD*) using Equation (5).

(5) $$WPD_{i}=\prod_{j=1}^{r}d_{i}{}^{w_{j}}$$

*Step 4:* Determine the relative importance score (*RIS*) of each experimental run using Equation (6) [34].

(6) $$RIS_{i}=\lambda \cdot WSD_{i}+\left(1-\lambda \right)\cdot WPD_{i}$$

where *λ* is a constant with a minimum value of 0 and a maximum value of 1, however in the reported studies, a value of 0.5 is proposed for good accuracy [35,36]. The highest *RIS* value is the best experimental run.

*Step 5:* Finally, the optimal parameter settings are obtained considering the average values of the *RIS* for each process parameter at each level. Higher average values of *RIS* represent better response performances.

## 4. Results and Discussion

### 4.1. Regression Models for Dimensional Deviation

Regression models computed for dimensional deviation in uncoded units are given in Equations (A1)–(A4) in Appendix B. The adequacy of these models is assessed based on the coefficient of determination (R^2^), adjusted R^2^, predicted R^2^, and lack of fit. Table 3 is the summary of dimensional deviations. The results show that the developed regression models are adequate and fit well with the experimental data due to their higher R^2^ values, which are near 100%, and their *p*-values are larger than the alpha value of 0.05. Further, the models have good prediction accuracy, as the adjusted R^2^ and predicted R^2^ are closer to each other (the percentage difference is less than 20%).

**Figure 5:**
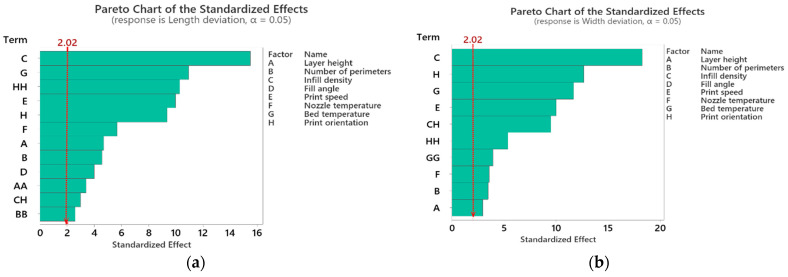

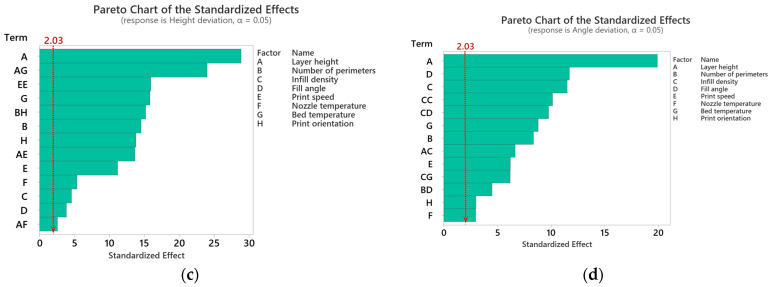
Pareto chart of standardized effect of dimensional deviation (**a**) length deviation, (**b**) width deviation, (**c**) height deviation, (**d**) angle deviation.

The effect of the process parameters on individual responses was studied through the Pareto chart for standardized effect. Figure 5a–d show that all the terms that crossed the reference line at 2.02 are significant at an alpha value of 0.05. Figure 5a illustrates that for length deviation, infill density is the most influential factor, followed by bed temperature, quadratic effect of print orientation, print speed, print orientation, nozzle temperature, layer height, number of perimeters, fill angle, quadratic effect of layer height, interaction of infill density and print orientation, and quadratic effect of the number of perimeters. Accordingly, for width deviation, as shown in Figure 5b, the most influential factors are infill density followed by print orientation, bed temperature, print speed, interaction of infill density and print orientation, quadratic effect of a number of perimeters, quadratic effect of bed temperature, nozzle temperature, number of parameters, and layer height. For height deviation, as shown in Figure 5c, the most influential factors include layer height, interaction of layer height, square of print speed, bed temperature, interaction of the number of perimeters and print orientation, number of perimeters, print orientation, interaction of layer height and print speed, print speed, nozzle temperature, infill density, fill angle and interaction of layer height, and nozzle temperature. For angle deviation, as shown in Figure 5d, the most influential factors are layer height, fill angle, infill density, square of infill density, interaction of infill density and fill angle, bed temperature, number of perimeters, interaction of layer height and infill density, print speed, interaction of infill density and bed temperature, number of perimeters and fill angle, print orientation, and nozzle temperature.

### 4.2. Main Effect and Interaction Plots

Main effect and interaction plots are generated for the modeled process parameters to study the effect of process parameters on dimensional deviations i.e., length deviation, width deviation, height deviation, and angular deviation.

#### 4.2.1. Main Effect and Interaction Effect of Process Parameters on Length Deviation

**Figure 6:**
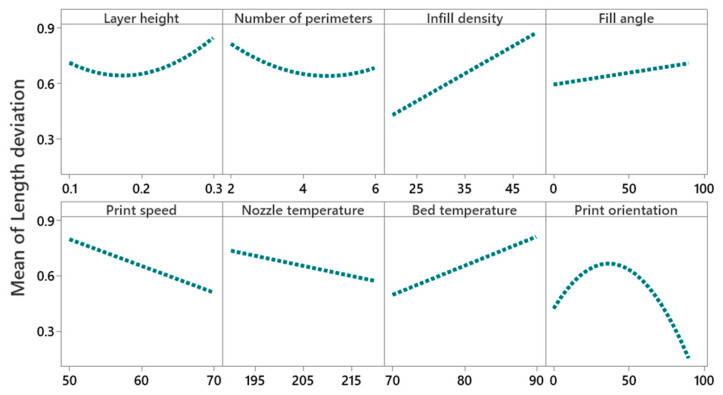
Main effect plot for length deviation.

**Figure 7:**
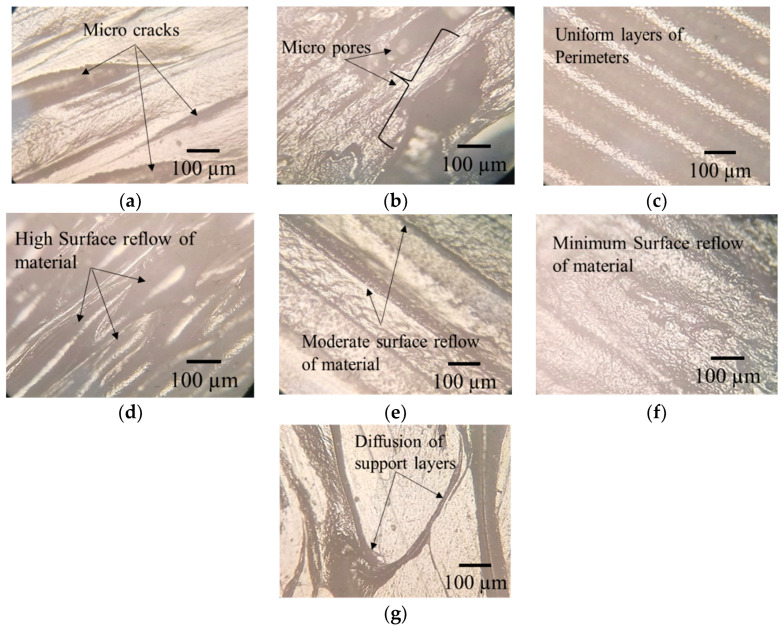
Optical images (**a**) formation of microcracks at 0.3 mm layer height, (**b**) formation of micropores at 0.3 mm layer height, (**c**) uniform layers of perimeters, (**d**) high surface reflow of material at 90 °C bed temperature, (**e**) moderate surface reflow of material at 80 °C bed temperature, (**f**) minimum surface reflow of material at 70 °C bed temperature, (**g**) diffusion of support layers at 45° build orientation.

The main effect plot given in Figure 6 shows that with an increase of the layer height from 0.1 mm to 0.2 mm, the mean length deviation decreases from 0.7% to 0.6%, and then it increases from 0.6% to 0.8%, with an increase of layer height from 0.2 mm to 0.3 mm. These results are in line with the findings of Deswal et al. [8], Agarwal et al. [37], and Nancharaiah et al. [38]. A high layer causes the formation of an air gap between the layers that reduces interlayer bonding and results in inner stresses that cause deformation and distortion of layers [24]. The cooling time of the material also decreases with an increase of the layer thickness, affecting the adhesion between layers. This increases dimensional deviation and also reduces the mechanical properties [24,39]. The optical inverted metallurgical microscope (Model No: M-41X, Lab Testing Technology Shanghai Co., Ltd., Shanghai, China) images in Figure 7a,b further confirms these conclusions. Its shows the formation of cracks and pores in printed parts at a layer height of 0.3 mm.

An increase of the number of perimeters from two to six decreases the deviation in length from 0.8% to 0.6%. According to Mohamed et al. [40], an increase of the number of perimeters increases the dimensional accuracy of the part length, as they are built parallel to the length [29]. A larger number of contours provide a dense filling in parts and make the part structure uniform with low dimensional deviation, as shown in Figure 7c.

**Figure 8:**
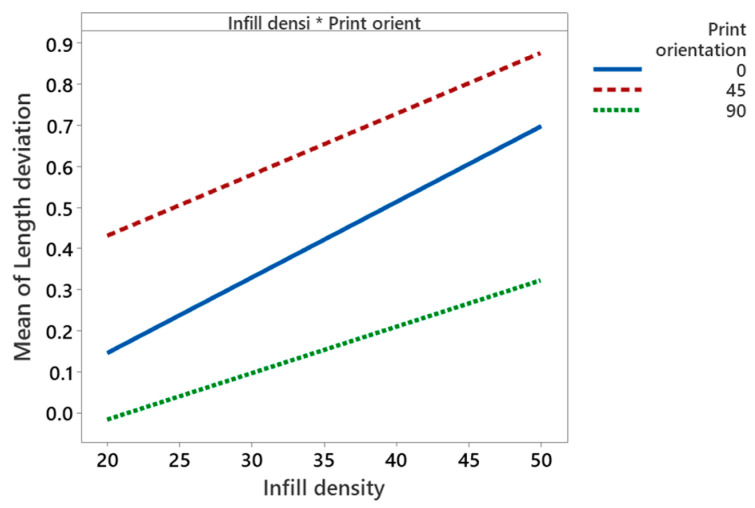
Interaction plot for length deviation.

Increase of infill percentage from 20% to 50% increases length deviation by 0.4% to 0.9%. These findings are in line with Akande et al. [41] and Agarwal [37]. A low infill density helps in transferring heat and cools down the material from glass transition temperature to ambient temperature without creating thermal stresses [8]. The interaction of infill density and the print orientation is found to be significant, as shown in Figure 8. It shows that the length deviation is minimal at lower infill density (20%) and higher print orientation (90°). At 90° print orientation, the mean width deviation is much lower for all values of infill density compared to other orientations.

An increase of the fill angle from 0° to 90° increases the length deviation by 0.6% to 0.75%. This may be due to the staircase effect that increases with an increase of raster angle [29]. At higher raster angles, voids are formed between the deposited raster and the perimeter walls causing incomplete filling and weak interlayer bonding which results in distortion and causes dimensional inaccuracies [40,42].

Increase of printing speed from 50 mm/s to 70 mm/s decreases length deviation by 0.8% to 0.5%. The same results were concluded by Agarwal et al. [37]. Low print speed allows more time for the deposition of material, and therefore, increases the dimensional deviation [27]. Generally, the polymer expands upon extrusion, however, by increasing the nozzle speed, the shear rate of polymer increases as a material is dragged by the nozzle tip and bed, thereby reducing the width of filament [43,44]. According to Brydson [45], by increasing the shear rate beyond a certain critical value, the extrusion swell decreases.

Length deviation decreases by 0.7% to 0.6% with an increase of nozzle temperature from 190 °C to 220 °C. High nozzle temperature maintains a consistent flow (good fluidity) of material that improves the fusion between layers and reduces the air gap, which helps in reducing distortion [46]. According to Afonso et al. [47], at an extrusion temperature between 210 °C to 230 °C, the PLA material becomes thermally and rheologically stable, and provides a good bonding mechanism between layers through a reduction of mesostructure voids, thereby improving the dimensional accuracy of printed parts.

An increase of the bed temperature from 70 °C to 90 °C increases the length deviation by 0.4% to 0.8%. This may be attributed to the glass transition temperature of PLA at about 60 °C. Near glass transition temperature, the mobility of macromolecules is higher, which improves the diffusion of polymer onto the glass and increases the adhesive forces [48]. According to Spoerk et al. [49], in the PLA material, adhesive forces increase with an increase of bed temperature, causing the bending of parts and damaging the bed surface upon cooling. Figure 7d shows a high layer diffusion and surface reflow of material, which results in dimensional deviations. However, it is moderate to minimum at bed temperatures of 80 °C and 70 °C, as illustrated in Figure 7e,f.

Length deviation increases by 0.4% to 0.7% with the increase of the build orientation from 0° to 45°, and then decreases by 0.7% to 0.1% from 45° to 90°. This is in line with the finding of Abdelrhman et al. [50]. This could be due to the diffusion of support material with part-built layers, which increases the surface roughness and induces dimensional inaccuracies, as shown in Figure 7g. The increase of length deviation may also be attributed to an increase of the staircase effect along the inclined surface (up to 45°), while the staircase effect is reduced by increasing the build orientation from 45° to 90° [31].

#### 4.2.2. Main Effect and Interaction Effect of Process Parameters for Width Deviation

**Figure 9:**
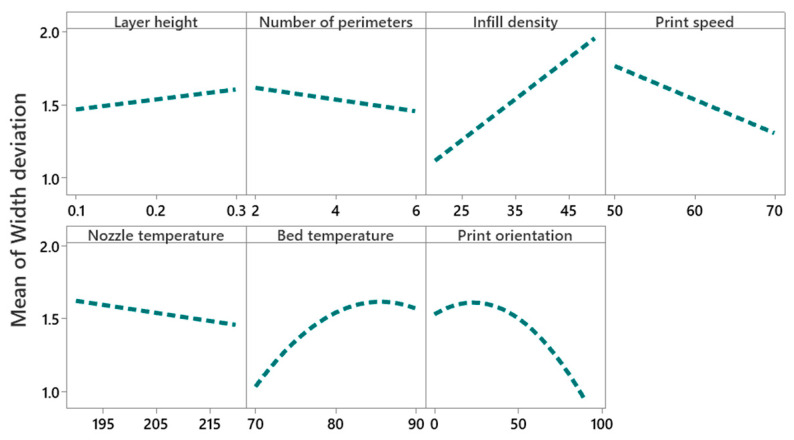
Main effect plot for width deviation.

**Figure 10:**
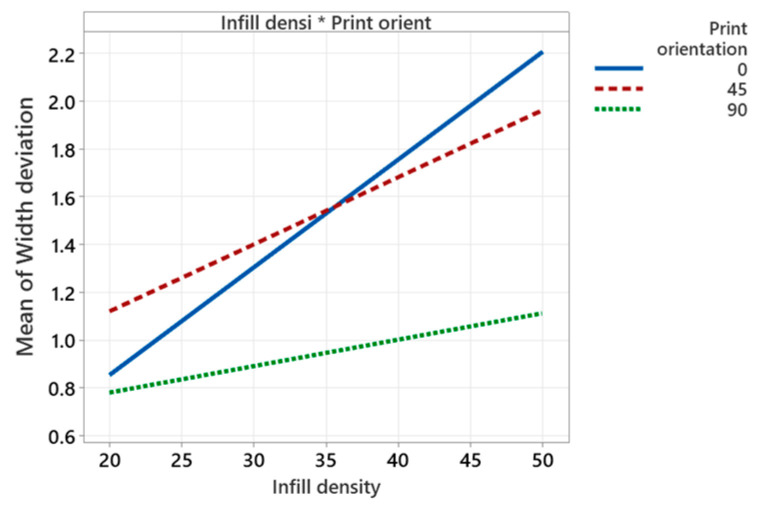
Interaction plot for width deviation.

Figure 9 shows that the mean width deviation increases (from 1.45% to 1.6%) with the increase of the layer height. High layer height causes an uneven temperature gradient along the built axis, which causes inner residual stresses and results in distortion of the layer [51]. Increasing the number of perimeters reduces the width deviation (from 1.6% to 1.5%), while a larger number of perimeters provide dense filling in parts and make the part structure uniform [40]. Increasing the infill density increases the width deviation significantly (from 1.1% to 2%) compared to the layer height and the number of perimeters. Further, its interaction with print orientation is also found to be significant, as explained in Figure 10. It shows that at an infill density of 20% and print orientation of 90°, the minimum mean width deviation is 0.8%; at 0° it is approximately 0.85%, and at 45° it is higher i.e., approximately 1.8%. Further, it illustrates that at 90° print orientation, the mean width deviation is much lower for all values of infill density compared to 0° and 45° print orientations. An increase of print speed decreases the mean width deviation from 1.8% to 1.4%. However, increasing the print speed beyond some critical value decreases the cooling cycle of deposited materials and causes a thermal gradient that results in poor dimensional accuracies [52]. Increasing nozzle temperature reduces the mean width deviation from 1.6% to 1.4%. With an increase of extrusion temperature, polymer viscosity reduces and facilitates a good deposition process with reduced voids due to a greater flow of material through the nozzle tip [53]. At low extrusion temperatures, the layers are not completely fused, and cracks and pores are produced between each layer, which causes stress concentration near the pores and affects the mechanical and dimensional accuracy of the part [53]. An increase of bed temperature significantly increases the mean width deviation from 1% to 1.6%. According to Srinivas et al. [18], in FDM printing, the degree of crystallinity is higher in the bottom layers than in the top and side layers because of bed temperature, which causes the layer to cool down slowly, resulting in dimensional inaccuracies. Benwood et al. [54] reported that a bed temperature of 90 °C increases the crystallinity of PLA printed parts to a greater extent, thereby increasing its mechanical strength. However, the high degree of crystallinity causes poor dimensional accuracies due to shrinkage and residual stresses [55].

#### 4.2.3. Main Effect and Interaction Effect of Process Parameters for Height Deviation

**Figure 11:**
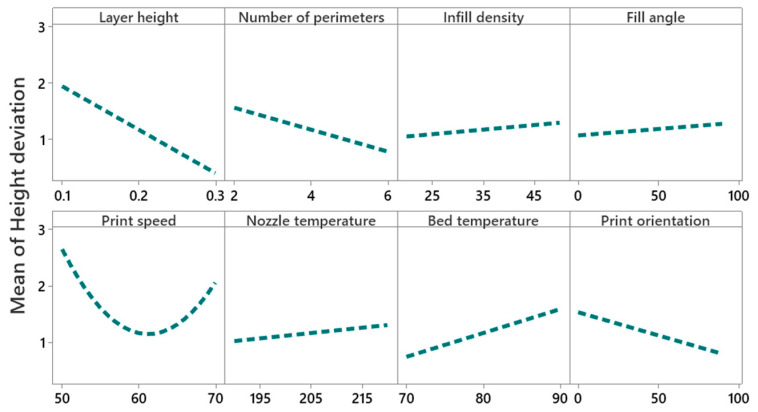
Main effect plot for height deviation.

**Figure 12:**
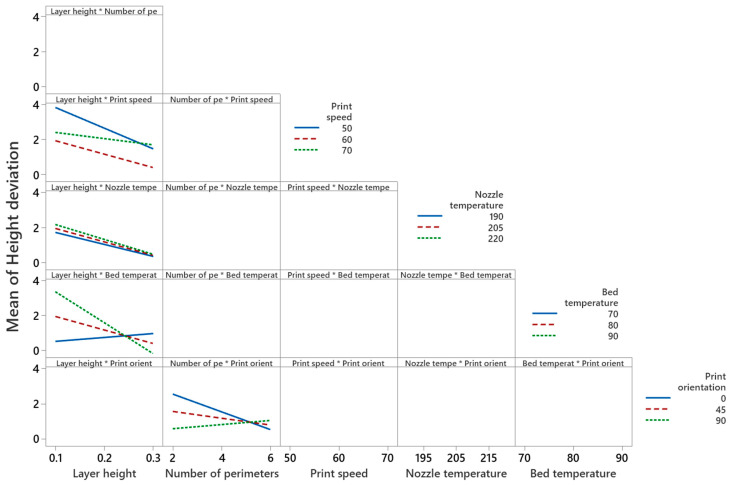
Interaction plot for height deviation.

Figure 11 shows that the mean height deviation decreases significantly (i.e., 2% to 0.2%) with the increase of layer height from 0.1 to 0.3 mm. These results are in line with studies by Deswal et al. [8], Camposeco-Negrete [56], and Peng et al. [57], which report that a high layer height reduces the deviation in thickness or the height of printed parts. The effect of layer height on mean height deviation is not sufficient information to interpret the results, due to the significant interaction between layer height and print speed, nozzle temperature, and bed temperature, as shown in the interaction plots in Figure 12. Figure 12 illustrates that at 60 mm/s print speed and 0.3 mm layer height, a minimum height deviation of 0.5% can be achieved. An interaction plot of layer height and nozzle temperature shows that at 0.3 mm layer height, a nozzle temperature of 190 °C minimizes the height deviation to 0.4%. Interaction plots of layer height and bed temperature prove that a bed temperature of 90 °C and a layer height of 0.3 mm reduces the height deviation to 0.09%. The interaction plot of the number of perimeters and print orientation depicts that a higher number of perimeters (i.e., six) and low print orientation (i.e., 0°) results in a minimum height deviation of 0.5%. The main effect plot of infill density and fill angle shows that the height deviation increases with an increase of infill density from 1% to 1.3%, and from 1% to 1.2% for fill angle.

#### 4.2.4. Main Effect and Interaction Effect of Process Parameters for Angle Deviation

**Figure 13:**
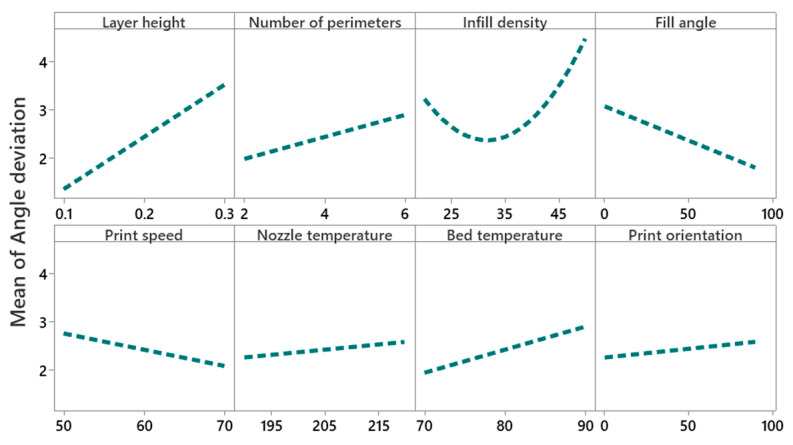
Main effect plot for angular deviation.

**Figure 14:**
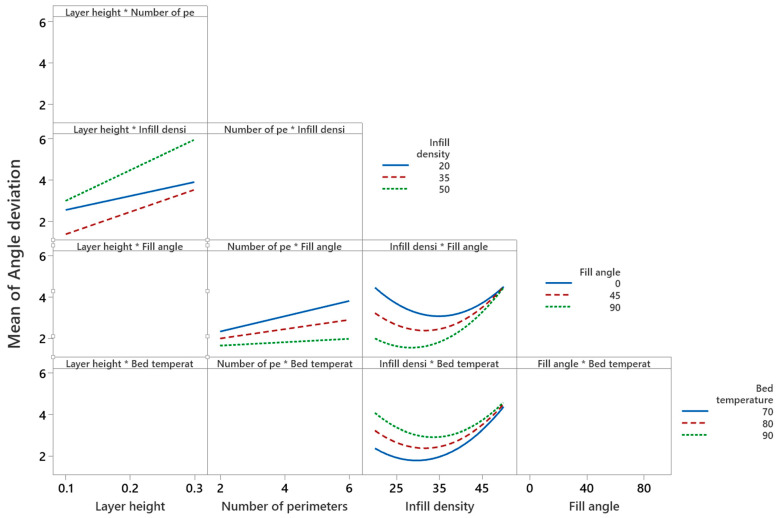
Interaction plot for angular deviation.

**Figure 15:**
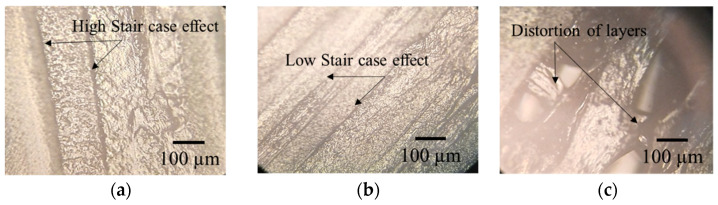
Optical microscope images (**a**) high staircase effect at 0.3 mm layer height, (**b**) high staircase effect at 0.1 mm layer height, (**c**) distortion of layers due to thermal stresses at 0.3 mm layer height and 50% infill density.

Figure 13 illustrates that the mean angular deviation increases with an increase of layer height. However, the interaction of layer height with infill density (as shown in Figure 14) implies that an infill density of 35% (medium level) and layer height of 0.1 mm (low level) give minimum angular deviation compared to 20% (low level) and 50% (elevated level) infill densities. The interaction plot of the number of perimeters and infill angle proves that the number of perimeters at a low level (i.e., two) and infill angle at a high level (i.e., 90°) reduces the angular deviation (i.e., from 2% to 1.5%). This may be due to the staircase effect that is more prominent in higher layer heights compared to lower layer heights, as shown in Figure 15a,b. Interaction plots of infill density with fill angle show that lower infill density (between 25% and 35%) and higher fill angle (90°) minimize the angular deviation. Figure 15c shows the thermal distortion of layers at high layer height and high infill density. An interaction plot of infill density with bed temperature proves that a bed temperature of 80 °C and infill density of 30% reduces the angular deviation to 1.7%. With an increase of print speed, the mean angular deviation decreases from 2.8% to 2%. However, with the increase of nozzle temperature, mean angular deviation increases from 2.2% to 2.4%, and a similar trend is seen for the print orientation.

### 4.3. Optimization

For individual response optimization, the desirability function is used. Equation (2) is applied to optimize the dimensional deviations i.e., the length deviation (DL), width deviation (WD), height deviation (HD), and angle deviation (AD) are minimized. The desirability values computed after optimization are tabulated in Table A2 and given in Appendix A. The optimal setting of process parameters was identified by calculating the average desirability values of each process parameter at each level, as shown in Table 4. The highest average desirability value denotes the best levels for the process parameters. Aimed at length deviation, the highest average desirability values computed for the layer height and the number of perimeters is 0.76 and 0.75 at level 0, respectively. For infill density, fill angle, and bed temperature, the values are 0.85, 0.75, and 0.80 at level −1, respectively. For print speed, nozzle temperature, and print orientation, the values are 0.61, 0.77, and 0.82 at level 1, respectively. Thus, the optimal settings to minimize the length deviation are calculated. Similar optimal settings were obtained for width deviation. The optimal setting for height deviation is layer height, number of perimeters and print orientation at level 1, infill density, fill angle, print speed, nozzle temperature at level 0, and bed temperature at level −1. The optimal setting for angle deviation is layer height, number of perimeters, nozzle temperature, and print orientation at level 1, infill density and bed temperature at level 0, and fill angle at level 1.

The optimal levels vary for the individual responses; therefore, it is important to perform simultaneous optimization of responses. An integrated approach of desirability and weighted aggregated sum product assessment (WASPAS) was implemented for multi-response optimization, as discussed in Section 3.3. Table 5 shows the optimal levels obtained based on the relative importance score (RIS). The RIS values are presented in Table A2. Higher mean values of the RIS at any level represent the best set of process parameters. The most optimal setting obtained for combined responses to reduce dimensional deviations are layer height, infill density, and bed temperature at level −1, having higher RIS mean values of 1.369, 1.578, and 1.545; the number of perimeters, fill angle, print speed nozzle temperature, and print orientation at level 1, with RIS mean values of 1.391, 1.425, 1.506, 1.374, and 1.535, respectively. The encoded values of these optimal settings are layer height at 0.1 mm, number of perimeters at six, infill density at 20%, fill angle at 90°, print speed at 70 mm/s, nozzle temperature at 220 °C, bed temperature at 70 °C, and print orientation at 90°.

Based on confirmatory experiments, the dimensional deviations obtained based on optimal settings are length deviation of 0.052%, width deviation of 0.086%, height deviation of 0.425%, and angle deviation of 0.211%.

## 5. Conclusions

One of the main challenges for 3D printing using fused deposition modeling (FDM) is the reduction of dimensional deviation. This is because of the numerous control parameters that must be considered in 3D printing. The present study analyzed and optimized the dimensional deviations, specifically in length deviation, width deviation, height deviation, and angle deviation. Based on experimental results and statistical analysis, the following conclusions are drawn:

The process parameters, including the layer height, number of perimeters, infill density, infill angle, print speed, nozzle temperature, bed temperature, and print orientation significantly affect the dimensional deviation. The most influential process parameters for length deviation are infill density followed by bed temperature and printing speed. For width deviation are infill density followed by print orientation and bed temperature. For height deviation are layer height followed by bed temperature and nozzle temperature. For angle deviation are layer height followed by fill angle and infill desnity.

An increase of the layer height from 0.1 to 0.3 mm causes an increase of the length deviation, width deviation, and angle deviation. An increase of the number of the perimeter (2 to 6) decreases dimensional deviation, however, it increases the angle deviation. Length, width, and angle deviation decrease with an increase of the print speed, while height deviation first decreases (from 50 to 60 mm/s) and then shows an increasing trend (from 60 to 70 mm/s). An increase of the fill angle from 0 to 90° increases the length, width, and height deviation, while it decreases the angle deviation. For print orientation from 0 to 45°, an increasing trend is observed for length deviation, while from 45 to 90°, it shows a decreasing trend.

From the obtained results, a definitive screening design was found for an efficient approach to model the non-linear quadratic models for dimensional deviation in smaller experimental runs.

The optimal settings for length deviation, width deviation, height deviation, and angle deviation vary according to the PLA material used. For length deviation, the optimal settings are layer height at 0.2 mm, number of perimeters at 4, infill angle at 0°, infill density at 25%, print speed at 70 mm/s, nozzle temperature at 220 °C, bed temperature at 70 °C, and print orientation at 90°. Similar optimal settings are obtained for width deviation. The optimal setting for the height deviation is layer height at 0.3 mm, number of perimeters at six, infill angle at 45°, infill density at 35%, print speed at 60 mm/s, nozzle temperature at 205 °C, bed temperature at 190 °C, and print orientation at 90°. For angle deviation, the optimal settings are layer height at 0.1 mm, number of perimeters at two, infill angle at 45°, infill density at 50%, print speed at 60 mm/s, nozzle temperature at 190 °C, bed temperature at 80 °C, and print orientation at 0°.

According to the proposed integrated approach of desirability and weighted aggregated sum product assessment (WASPAS), the optimal settings are layer height at 0.1 mm, number of perimeters at six, infill density at 20%, fill angle at 90°, print speed at 70 mm/s, nozzle temperature at 220 °C, bed temperature at 70 °C, and print orientation at 90°. The dimensional deviations based on these optimal settings are length deviation of 0.052%, width deviation of 0.086%, height deviation of 0.425%, and angle deviation of 0.211%.

This study provides a guideline for the practitioner to choose the right set of FDM printing process parameters.

In future work, the optimized results of the current study will be utilized for fabricating assistive devices that need control dimensions, including hand orthoses and foot orthoses.

## Figures and Tables

**Table 1:** Levels for printing parameters.

Printing Parameters	Symbol	Units	Levels		
			−1	0	1
Layer Height	A	mm	0.1	0.2	0.3
Number of Perimeters	B	-	2	4	6
Infill density	C	%	20	35	50
Fill angle	D	°	0	45	90
Print Speed	E	mm/s	50	60	70
Nozzle temperature	F	°C	190	205	220
Bed temperature	G	°C	70	80	90
Print orientation	H	°	0	45	90

**Table 2:** Printing parameters that are kept constant.

Printing Parameters	Settings
Pattern type	Rectilinear
Solid layers	3 for both top and bottom
Air gap	Negative
First layer speed	20 mm/s
Retraction speed	100/s

**Table 3:** Models summary of dimensional deviation.

Responses	R^2^ (%)	Adjusted R^2^ (%)	Predicted R^2^ (%)	Lack of Fit Based on *p*-Value
LD (%)	95.36	93.89	91.2	0.848
WD (%)	95.71	94.64	92.96	0.899
HD (%)	98.74	98.3	97.5	0.901
AD (%)	97.18	96.19	94.51	0.545

**Table 4:** Optimal levels for individual responses.

Process Parameters	Levels			Optimal Levels of Responses			
	−1	0	1	DL	DW	DH	DA
	DL, DW, DH, DA						
Layer Height	0.72, 0.61, 0.51, 0.68	0.76, 0.64, 0.57, 0.58	0.66, 0.55, 0.84, 0.40	0	0	1	−1
Number of Perimeters	0.65, 0.55, 0.65, 0.62	0.75, 0.63, 0.47, 0.56	0.73, 0.62, 0.75, 0.49	0	0	1	−1
Infill density	0.85, 0.77, 0.65, 0.62	0.68, 0.58, 0.70, 0.52	0.57, 0.42, 0.65, 0.42	−1	−1	0	0
Fill angle	0.75, 0.62, 0.66, 0.47	0.67, 0.50, 0.67, 0.52	0.68, 0.61, 0.65, 0.66	−1	−1	0	1
Print Speed	0.61, 0.52, 0.59, 0.48	0.67, 0.49, 0.75, 0.68	0.81, 0.71, 0.69, 0.57	1	1	0	0
Nozzle temperature	0.67, 0.59, 0.67, 0.59	0.66, 0.49, 0.68, 0.50	0.77, 0.64, 0.61, 0.45	1	1	0	−1
Bed Temperature	0.80, 0.71, 0.72, 0.60	0.70, 0.56, 0.68, 0.72	0.61, 0.49, 0.60, 0.45	−1	−1	−1	0
Print orientation	0.66, 0.49, 0.62, 0.59	0.54, 0.48, 0.65, 0.49	0.82, 0.75, 0.71, 0.55	1	1	1	−1

**Table 5:** Optimal levels for combined responses based on the grey relational grade.

Process Parameters	Levels			Optimal Levels
	−1	0	1	
Layer Height	1.369	1.360	1.323	−1
Number of Perimeters	1.346	1.254	1.391	1
Infill density	1.578	1.463	1.069	−1
Fill angle	1.314	1.249	1.425	1
Print Speed	1.157	1.426	1.506	1
Nozzle temperature	1.365	1.248	1.374	1
Bed Temperature	1.545	1.431	1.116	−1
Print orientation	1.243	1.157	1.535	1

## Data Availability

The data presented in this study are available on request from the corresponding author.

## Appendix A

**Table A1:** Experimental design and measured responses.

Exp. No.	A	B	C	D	E	G	H	I	Length Deviation (LD)	Width Deviation (WD)	Height Deviation (HD)	Angle Deviation (AD)
1	0.2	6	50	90	70	220	90	90	0.273	0.600	2.900	4.556
2	0.1	2	50	90	70	205	70	0	0.618	1.360	2.600	2.333
3	0.3	6	20	90	70	190	80	0	0.309	0.680	1.200	2.156
4	0.1	6	35	90	50	190	70	90	0.182	0.400	2.000	0.644
5	0.3	6	50	45	50	220	70	0	0.836	1.840	1.300	6.867
6	0.3	6	20	0	50	205	90	90	0.491	1.080	0.400	7.511
7	0.3	6	20	90	70	190	80	0	0.545	0.804	0.800	2.289
8	0.1	2	50	0	50	220	80	90	0.600	1.320	3.500	2.867
9	0.3	2	20	90	60	220	70	90	0.164	0.360	0.200	2.067
10	0.3	6	20	0	50	205	90	90	0.455	1.000	0.700	7.067
11	0.1	2	50	0	50	220	80	90	0.673	1.480	3.600	2.778
12	0.1	6	50	0	60	190	90	0	1.036	2.280	2.400	2.844
13	0.1	2	20	45	70	190	90	90	0.218	0.480	2.600	2.400
14	0.1	6	20	0	70	220	70	45	0.055	0.120	0.618	3.800
15	0.1	6	20	0	70	220	70	45	0.073	0.160	0.636	4.333
16	0.1	2	50	90	70	205	70	0	0.691	1.520	2.500	2.044
17	0.1	6	50	0	60	190	90	0	0.964	2.120	2.700	4.111
18	0.3	2	35	0	70	220	90	0	0.673	1.480	2.400	3.600
19	0.1	6	20	0	70	220	70	45	0.091	0.200	0.588	2.311
20	0.1	2	50	0	50	220	80	90	0.473	1.040	3.400	3.067
21	0.1	2	20	45	70	190	90	90	0.273	0.600	2.900	2.489
22	0.1	6	35	90	50	190	70	90	0.473	0.674	2.400	0.489
23	0.2	4	35	45	60	205	80	45	0.673	1.480	1.109	2.333
24	0.1	4	20	90	50	220	90	0	0.473	1.040	6.000	2.867
25	0.3	2	35	0	70	220	90	0	0.582	1.280	2.700	3.511
26	0.3	6	20	90	70	190	80	0	0.345	0.760	1.000	1.956
27	0.2	2	20	0	50	190	70	0	0.309	0.680	3.100	3.133
28	0.1	4	20	90	50	220	90	0	0.273	0.925	5.700	2.600
29	0.3	6	50	45	50	220	70	0	0.782	1.720	1.500	6.556
30	0.1	2	50	90	70	205	70	0	0.745	1.640	2.800	2.267
31	0.2	4	35	45	60	205	80	45	0.709	1.560	1.055	2.711
32	0.2	2	20	0	50	190	70	0	0.273	0.600	3.400	2.689
33	0.1	6	50	0	60	190	90	0	0.927	2.040	2.500	3.600
34	0.3	2	50	90	50	190	90	45	1.782	2.720	1.455	5.689
35	0.3	6	50	45	50	220	70	0	0.873	1.920	1.900	6.111
36	0.3	4	50	0	70	190	70	90	0.200	0.440	1.700	5.511
37	0.3	4	50	0	70	190	70	90	0.091	0.200	1.900	5.667
38	0.2	6	50	90	70	220	90	90	0.345	0.760	2.500	4.933
39	0.3	2	50	90	50	190	90	45	1.673	2.480	1.436	6.267
40	0.1	4	20	90	50	220	90	0	0.673	1.008	5.800	2.400
41	0.2	6	50	90	70	220	90	90	0.382	0.840	2.800	4.778
42	0.3	2	35	0	70	220	90	0	0.636	1.400	2.300	3.422
43	0.1	6	35	90	50	190	70	90	0.382	0.840	2.100	0.978
44	0.2	2	20	0	50	190	70	0	0.345	0.760	3.300	3.000
45	0.3	2	50	90	50	190	90	45	1.545	2.320	1.473	6.111
46	0.1	2	20	45	70	190	90	90	0.418	0.920	3.100	2.533
47	0.2	4	35	45	60	205	80	45	0.636	1.400	1.200	2.511
48	0.3	6	20	0	50	205	90	90	0.436	0.960	0.600	7.222
49	0.3	2	20	90	60	220	70	90	0.218	0.480	0.400	1.822
50	0.3	4	50	0	70	190	70	90	0.400	0.880	2.000	5.867
51	0.3	2	20	90	60	220	70	90	0.109	0.240	0.700	1.933

***Calculation of integrated approach of desirability and weighted aggregated sum product assessment (WASPAS).***

In Table A2, weighted sum desirability (WSD) is calculated using Equation (4). Weighted product desirability (WPD) is computed based on Equation (5). In the present study, equal weights are considered so it is set at 1. WSD and WPD are combined using Equation (6) to get a relative importance score (RIS).

**Table A2:** Desirability and relative importance score values of responses.

Exp. No.	Desirability Values of Responses				Multi-Response Optimization		
	DL	WD	HD	AD	WSD	WPD	RIS
1	0.865	0.797	0.554	0.421	2.636	0.161	1.398
2	0.652	0.475	0.661	0.737	2.524	0.151	1.337
3	0.843	0.763	0.905	0.763	3.273	0.444	1.859
4	0.808	0.881	0.714	0.978	3.381	0.497	1.939
5	0.517	0.271	0.839	0.092	1.719	0.011	0.865
6	0.73	0.593	1	0	2.324	0	1.162
7	0.806	0.71	0.973	0.744	3.233	0.414	1.824
8	0.663	0.492	0.446	0.661	2.262	0.096	1.179
9	0.933	0.898	0.991	0.775	3.597	0.644	2.12
10	0.753	0.627	0.946	0.063	2.39	0.028	1.209
11	0.618	0.511	0.429	0.674	2.231	0.091	1.161
12	0.393	0.085	0.643	0.592	1.713	0.013	0.863
13	0.899	0.847	0.553	0.728	3.027	0.307	1.667
14	1	1	0.616	0.528	3.145	0.326	1.735
15	0.989	0.983	0.611	0.545	3.128	0.324	1.726
16	0.607	0.407	0.696	0.778	2.488	0.134	1.311
17	0.438	0.153	0.589	0.484	1.664	0.019	0.842
18	0.618	0.424	0.734	0.557	2.333	0.107	1.22
19	0.978	0.966	0.618	0.611	3.172	0.356	1.764
20	0.674	0.528	0.464	0.633	2.299	0.105	1.202
21	0.865	0.797	0.554	0.715	2.931	0.273	1.602
22	0.742	0.765	0.675	1	3.182	0.383	1.782
23	0.618	0.424	0.659	0.737	2.438	0.127	1.283
24	0.742	0.61	0	0.661	2.013	0	1.007
25	0.674	0.508	0.759	0.57	2.511	0.148	1.33
26	0.82	0.729	0.938	0.791	3.278	0.443	1.861
27	0.843	0.763	0.518	0.623	2.747	0.208	1.477
28	0.736	0.659	0.054	0.699	2.147	0.018	1.083
29	0.551	0.322	0.804	0.136	1.812	0.019	0.916
30	0.573	0.356	0.643	0.747	2.319	0.098	1.208
31	0.596	0.39	0.645	0.684	2.314	0.102	1.208
32	0.865	0.797	0.464	0.687	2.813	0.22	1.516
33	0.461	0.186	0.625	0.557	1.829	0.03	0.929
34	0.017	0.011	0.68	0.259	0.967	0	0.484
35	0.494	0.237	0.795	0.199	1.726	0.019	0.872
36	0.91	0.864	0.768	0.285	2.827	0.172	1.5
37	0.934	0.828	0.732	0.263	2.757	0.149	1.453
38	0.82	0.729	0.625	0.367	2.541	0.137	1.339
39	0	0	0.684	0.177	0.861	0	0.43
40	0.736	0.624	0.036	0.728	2.123	0.012	1.067
41	0.798	0.695	0.571	0.389	2.453	0.123	1.288
42	0.64	0.458	0.777	0.582	2.457	0.133	1.295
43	0.798	0.794	0.696	0.93	3.218	0.41	1.814
44	0.82	0.729	0.482	0.642	2.674	0.185	1.429
45	0.03	0.068	0.666	0.199	0.963	0	0.481
46	0.839	0.753	0.518	0.709	2.819	0.232	1.526
47	0.64	0.458	0.652	0.712	2.462	0.136	1.299
48	0.764	0.644	0.964	0.041	2.414	0.02	1.217
49	0.899	0.847	1	0.81	3.556	0.617	2.087
50	0.885	0.827	0.714	0.234	2.661	0.122	1.392
51	0.966	0.949	0.978	0.794	3.687	0.712	2.2

## Appendix B

Regression equations for length deviation (LD), width deviation (WD), height deviation (HD) and angle deviation (AD)

(A1) $$\mathrm{LD}\left(\%\right)=1.340-4.450\;A\;-0.229\;B+0.015\;C\;+0.0013\;D\;-0.0144\;E\;-0.00545+0.0158\;G\;+0.0161H\;+12.82{\;A}^{2}+0.0245{\;B}^{2}-0.000181{\;H}^{2}-0.000079\;C\cdot H$$

(A2) $$\mathrm{WD}\left(\%\right)=-11.05+0.685\;A\;-0.0400\;B+0.04502\;C\;-0.0231\;E\;-0.0055\;F+0.3598\;G+0.02016\;H\;-0.002378{\;G}^{2}-0.00015{\;H}^{2}-0.00038\;C\cdot H$$

(A3) $$\mathrm{HD}\left(\%\right)=33.29+48.40\;A\;-0.5065\;B+0.00813\;C\;+0.002286\;D\;-1.5427\;E+0.02070\;F\;\;+0.24078\;G-0.03596\;H\;+0.011916{\;E}^{2}+0.4148\;A\cdot E\;-0.0560\;A\cdot F-0.9930\;A\cdot G\;+0.006939\;B\cdot H$$

(A4) $$\mathrm{AD}\left(\%\right)=-0.87+1.38\;A\;+0.3716\;B-0.3166\;C\;-0.03263\;D-0.03370\;E+0.01072\;F\;+0.1355\;G\;+0.00361\;H\;+0.006277{\;C}^{2}+0.2697\;A\cdot C-0.003207\;B\cdot D+0.000895\;C\cdot D\;-0.002505\;C\cdot G$$
